# Availability and Use of Cheap Tobacco in the United Kingdom 2002–2014: Findings From the International Tobacco Control Project

**DOI:** 10.1093/ntr/ntx108

**Published:** 2017-05-19

**Authors:** Timea R Partos, Anna B Gilmore, Sara C Hitchman, Rosemary Hiscock, J Robert Branston, Ann McNeill

**Affiliations:** 1 Addictions Department, King’s College London, London, United Kingdom; 2 UK Centre for Tobacco & Alcohol Studies, Nottingham, United Kingdom; 3 Department for Health, University of Bath, Bath, United Kingdom; 4 Centre for Governance and Regulation, School of Management, University of Bath, Bath, United Kingdom

## Abstract

**Introduction:**

Raising tobacco prices is the most effective population-level intervention for reducing smoking, but this is undermined by the availability of cheap tobacco. This study monitors trends in cheap tobacco use among adult smokers in the United Kingdom between 2002 and 2014 via changes in product type, purchase source, and prices paid.

**Methods:**

Weighted data from 10 waves of the International Tobacco Control policy evaluation study were used. This is a longitudinal cohort study of adult smokers with replenishment; 6169 participants provided 15812 responses. Analyses contrasted (1) *product type*: roll-your-own (RYO) tobacco, factory-made packs (FM-P), and factory-made cartons (FM-C); (2) *purchase source*: UK store-based sources (e.g., supermarkets and convenience stores) with non-UK/nonstore sources representing tax avoidance/evasion (e.g., outside the UK, duty free, and informal sellers); and (3) *prices paid* (inflation-adjusted to 2014 values). Generalized estimating equations tested linear changes over time.

**Results:**

(1) RYO use increased significantly over time as FM decreased. (2) UK store-based sources constituted approximately 80% of purchases over time, with no significant increases in tax avoidance/evasion. (3) Median RYO prices were less than half that of FM, with FM-C cheaper than FM-P. Non-UK/nonstore sources were cheapest. Price increases of all three product types from UK store-based sources from 2002 to 2014 were statistically significant but not substantial. Wide (and increasing for FM-P) price ranges meant each product type could be purchased in 2014 at prices below their 2002 medians from UK store-based sources.

**Conclusions:**

Options exist driving UK smokers to minimize their tobacco expenditure; smokers do so largely by purchasing cheap tobacco products from UK stores.

**Implications:**

The effectiveness of price increases as a deterrent to smoking is being undermined by the availability of cheap tobacco such as roll-your-own tobacco and cartons of packs of factory-made cigarettes. Wide price ranges allowed smokers in 2014 to easily obtain cigarettes at prices comparable to 12 years prior, without resorting to tax avoidance or evasion. UK store-based sources accounted for 80% or more of all tobacco purchases between 2002 and 2014, suggesting little change in tax avoidance or evasion over time. There was a widening price range between the cheapest and most expensive factory-made cigarettes.

## Introduction

Raising taxes to increase the price of tobacco is the most effective population-level intervention for reducing smoking^1,2^ and among the few policies shown to reduce inequalities in smoking.^3–6^ The World Health Organization (WHO) identifies price and tax measures as one of the key areas of tobacco control.^7^ The United Kingdom is leading the way, with real tobacco prices among the highest in the world.^8,9^ The potential public health benefits of tobacco tax increases are, however, influenced by a variety of factors including the availability of cheap tobacco and smokers’ purchasing choices. There is mounting evidence that smokers would be more responsive to price increases if there were fewer opportunities to obtain cheap tobacco.^1,10–12^ Disadvantaged smokers are more likely to use cheap tobacco,^13–16^ so its availability may also contribute to the widening socioeconomic disparities associated with smoking. The present study therefore aims to track cheap tobacco sources and use among adult smokers in the United Kingdom between 2002 and 2014 via changes in product type, purchase source, and prices paid and to identify the implications for tobacco tax policy.

Smokers can minimize their tobacco expenditure by changing the type of product they buy or the source from which they buy it. In terms of product type, smokers can change from more expensive factory-made (FM) cigarettes to cheaper roll-your-own (RYO) tobacco,^17,18^ change the brand they smoke (there is a large range in price between “premium” and “discount” brands^14,19^), or purchase in bulk (FM cigarettes are often cheaper purchased in bulk by the carton than by the single pack^20,21^). With regard to purchase source, smokers can purchase from supermarkets rather than convenience stores or from sources where duties are either minimized or not paid at all. The latter includes legal products (e.g., duty-free or from low tax jurisdictions outside the United Kingdom —commonly known as tax avoidance^22^), and illicit tobacco (including counterfeit and smuggled—commonly known as tax evasion^23^). The incentives for smokers to change their purchasing behaviors will depend on the price differences between, and ease of obtaining, the varying products.

In the United Kingdom during the study period of interest, changes occurred both in the rates of tobacco taxation and in the strategies adopted to curb illicit trade, so a rise in tobacco prices over time and a reduction in illicit trade were expected. From 2001 to 2008, tobacco taxation increased at the rate of inflation. In 2010, the UK government modified the tobacco tax structure, in part to combat the industry segmentation of the market into “premium” and “discount” sectors and also committed to keeping tobacco duty at least 2% above inflation from 2011 to 2014.^24,25^ In 2011, an additional 10% increase on RYO duty was also implemented.^24^ The UK tobacco duty rates from 2001 to 2014 are presented in Table 1. The first comprehensive strategy to tackle illicit tobacco in the United Kingdom was implemented in 2000 and included £201 million of targeted funding, 1000 new customs staff, a national network of freight scanners, the introduction of “UK duty paid” markings on all tobacco packs, harsher penalties for tobacco smuggling, cooperation with tobacco companies to reduce the availability of tobacco to smugglers, and an awareness-raising publicity campaign.^26^ This strategy was reinforced and updated in 2006 with the major change being an increased focus on RYO^27^ and also in 2011 where changes in European Union (EU) law allowed for tougher sanctions for illicit traders.^28^ A recent government review has credited these efforts with reducing the UK illicit tobacco market from 22% for FM and 61% for RYO tobacco in 2000, to 10% for FM and 39% for RYO tobacco in 2013/2014.^29^ In 2009, a program to tackle illicit tobacco in the north of England was also launched, which placed an emphasis on reducing the demand for illicit tobacco, and this was also evaluated as largely meeting its aims.^30^

**Table 1.** Tobacco duty rates for factory-made (FM) cigarettes and roll-your-own (RYO) tobacco in the United Kingdom from 2001 to 2014

	Specific duty^a^, £ per 1000 FM cigarettes	Specific duty^a^, £ per kilogram of RYO tobacco	Ad Valorem^b^ % (factory- made cigarettes only)	Value added tax (VAT)^c^ %	Relative to inflation^d^
2001 March	92.25	96.81	22.0	17.5	= inflation
2002 April	94.24	98.66	22.0	17.5	= inflation
2003 April	96.88	101.42	22.0	17.5	= inflation
2004 March	99.80	104.47	22.0	17.5	= inflation
2005 April	102.39	107.18	22.0	17.5	= inflation
2006 March	105.10	110.02	22.0	17.5	= inflation
2007 March	108.65	113.74	22.0	17.5	= inflation
2008 March	112.07	117.32	22.0	17.5	= inflation
2008 November	112.07	122.01	24.0	15.0	= inflation
2009 April	114.31	124.45	24.0	15.0	2% above
2010 March	119.03	129.59	24.0	17.5	1% above
2011 March	145.95	151.90	16.5	20.0	2% above
2012 March	167.41	164.11	16.5	20.0	5% above
2013 March	176.22	172.74	16.5	20.0	2% above
2014 March	184.10	180.46	16.5	20.0	2% above

^a^Specific duty is set in fixed cash terms as an amount per 1000 FM cigarettes or per kilogram of RYO tobacco.

^b^Ad Valorem duty is set as a percentage of the retail price, and is only applied to FM cigarettes.

^c^Value added tax (VAT) is set as a percentage of the retail price and is applied to all consumer goods.

^d^Data taken from Action on Smoking and Health (ASH) UK analysis of tobacco tax increases in the United Kingdom fact sheet.^24^

Availability and use of cheap tobacco is associated with reduced smoking cessation,^11–13,31^ underlining the importance of understanding the sources and types of cheap tobacco and the incentives underpinning their use. Research to date has indicated that RYO use in the United Kingdom is increasing,^18^ particularly among younger smokers,^32^ and between 2006 and 2009, the market share of discount FM brands increased significantly in the United Kingdom while their prices remained largely unchanged.^19^ In contrast, self-reported tax avoidance and evasion showed a declining trend among UK smokers from 2002 to 2011.^33^ Understanding the trends in cheap tobacco use is vital for informing tobacco control policy not least because the tobacco industry and its allies repeatedly argue that tax evasion is increasing in light of high tobacco taxes in the United Kingdom.^34,35^ With other countries looking to increase tobacco taxes, yet fearful of the potential impact on illegal sales and tax revenues, this article will be of importance further afield.

Prior studies examining price minimizing have focused on one particular aspect, or considered a limited time frame.^34^ The present study uses data from the International Tobacco Control (ITC) study^35,36^ to track cheap tobacco sources and use among adult smokers in the United Kingdom between 2002 and 2014. It does so by monitoring changes in product type, purchase source, and prices paid. The ITC is unique in making it possible to track concurrently multiple forms of price minimizing behavior in a single data set over a substantial period.

## Methods

### Participants

Data were from the first 10 waves (2002–2014) of the UK arm of the ITC project.^35,36^ This is a longitudinal cohort survey of adult smokers (18+) at recruitment with yearly replenishment (except at wave 8). Respondents who quit are also followed up. The survey uses a stratified random sample design and was administered either via computer-aided telephone interviewing or online (piloted in wave 7 and introduced gradually from wave 8 in 2010 onward). Surveys were conducted approximately annually, although some longer interwave intervals resulted in no surveys taking place in 2009, 2011, or 2012. Population cross-sectional sampling weights were calculated at each wave to be representative of national distributions of age, sex, and geographical region, and longitudinal weights were adjusted for attrition. Participants were included in the present analyses if they smoked at least monthly at the time of the survey and had smoked more than 100 cigarettes in their lifetime. Table 2 presents the demographic characteristics of the eligible study sample: *N* = 6169 participants who provided 15812 responses over the 10 waves. On average, each individual took part in 2.6 surveys (*SD* = 2.0).

**Table 2.** Unweighted sample characteristics by survey wave

	w1 2002	w2 2003	w3 2004	w4 2005	w5 2006	w6 2007	w7 2008	w8 2010	w9 2013	w10 2014
Met selection criteria, *N*	2367	1914	1831	1727	1690	1636	1474	960	1096	1117
Sex %										
Female	56.6	55.4	55.8	57.2	57.2	57.4	55.8	55.3	51.0	52.9
Male	43.4	44.6	44.2	42.9	42.8	42.6	44.2	44.7	49.0	47.1
Age brackets, years, %										
18–24	8.5	6.4	5.0	4.4	4.7	4.8	3.7	2.6	4.2	3.0
25–39	32.2	29.6	27.7	26.0	24.6	24.4	20.8	14.4	21.4	21.6
40–54	33.9	36.3	37.8	38.6	36.6	36.7	35.7	37.0	34.0	33.4
55+	25.4	27.6	29.5	31.0	34.1	34.2	39.8	46.0	40.3	42.1
Geographical region %										
London	13.4	13.3	12.3	12.1	13.3	13.1	11.5	11.7	10.3	10.8
Yorkshire and The Humber	8.8	8.6	8.6	8.9	8.1	7.1	6.7	7.0	7.5	7.6
East Midlands	6.8	7.0	7.7	7.1	7.0	7.3	8.0	7.2	6.7	6.8
Eastern	8.5	8.3	8.8	8.9	7.9	8.2	7.6	8.9	9.6	9.8
North East	4.7	4.6	4.6	5.0	4.9	4.6	4.3	4.1	4.3	4.5
South East	13.7	14.0	13.9	13.1	13.3	13.2	13.1	14.3	13.1	13.5
South West	7.7	8.1	8.0	8.1	8.4	8.3	9.4	8.4	7.9	8.2
West Midlands	8.5	8.9	8.7	8.1	7.7	8.7	8.6	8.3	9.5	8.0
North West	10.6	10.6	10.9	10.9	10.1	9.7	9.4	9.2	11.0	11.5
Wales	5.0	4.7	4.6	5.3	5.9	5.9	6.7	6.5	5.8	5.9
Scotland	9.9	10.0	10.2	10.1	10.9	10.7	11.5	12.0	11.0	10.7
Northern Ireland	2.3	2.0	2.5	2.5	2.6	3.2	3.2	2.5	3.5	2.8
Income brackets^a^, %										
Low	17.2	23.0	22.4	24.5	25.7	25.6	24.8	25.0	25.3	21.6
Moderate	44.1	39.0	39.7	40.6	40.6	38.7	38.2	37.5	36.7	37.8
High	29.2	29.7	29.6	26.7	24.8	25.7	27.0	28.5	30.2	32.3
Not disclosed	9.6	8.3	8.4	8.2	8.9	10.1	10.0	9.0	7.9	8.3
Missing/excluded data^b^, %										
Usual tobacco product	0.04	0.21	0.05	0.23	0.00	0.00	0.14	1.98	0.00	0.18
Product last purchased	2.15	2.09	0.82	0.69	0.47	3.73	0.95	4.48	2.55	2.24
Source of last purchase	0.38	0.52	0.11	0.12	0.24	0.79	0.54	0.21	1.51	1.52
Price	7.73	4.86	3.77	5.50	5.21	8.13	6.17	8.75	16.97	17.64
All valid (complete cases)	90.5	94.0	96.1	94.3	94.3	91.1	93.6	91.0	82.2	81.7

^a^Income brackets are based on annual household income, equivalized for household composition and Consumer Price Index (CPI) adjusted to 2014 values.

^b^Note that missing data + complete cases do not sum to 100%, as it was possible for participants to have data missing on more than one variable.

### Measures

#### Demographics

For descriptive purposes and missing data analyses, participants at each wave were asked their sex, age, annual household income, household composition, and geographical region. Household income was adjusted for household composition, converted to 2014 values using Consumer Price Index (CPI) data from the UK Office for National Statistics,^37^ and stratified to “low,” “medium,” and “high” values. Refusals to report income were retained as a separate category.

#### Cheap Tobacco Product Type and Purchase Source

**Figure 1.**
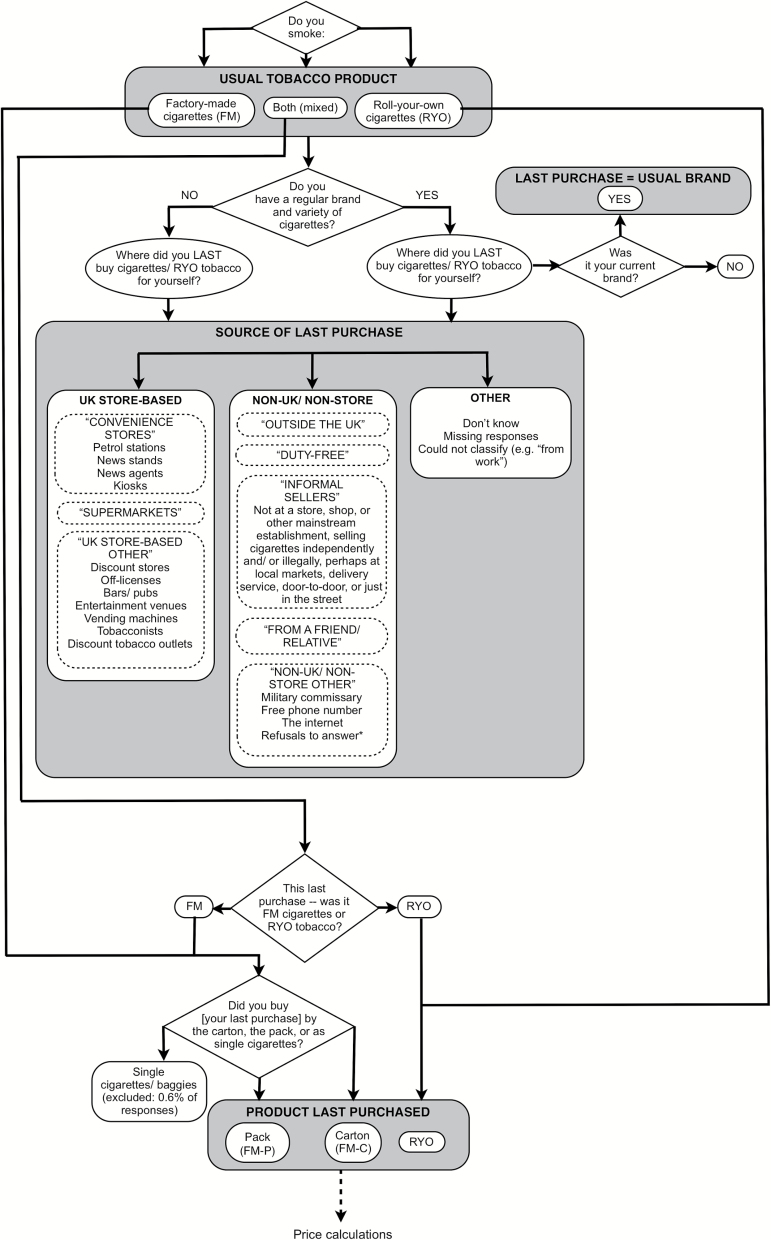
Schematic of survey questions and categorization of source and type of tobacco products. *Note*. The low reported prices associated with refusals to answer were consistent with this source being classified as “non-UK/nonstore”.

Figure 1 presents a schematic of the survey questions used to classify tobacco product types and purchase sources. Each participant’s usual tobacco product was determined by asking “Do you now smoke... (packet/factory-made cigarettes only; roll-your-own cigarettes only; both)?” All remaining indicators of cheap tobacco use were based on participants’ last reported tobacco purchase. These were classified as FM cigarettes by the *pack* (FM-P), FM cigarettes in a *carton* containing multiple packs (FM-C), or RYO tobacco. Smokers who indicated having a usual brand and variety of tobacco (see Figure 1) were asked if their last purchase was their usual brand. Buying nonusual brands may indicate being less brand-loyal or more swayed by in-store discounts and price promotions.

A novel approach to classifying tobacco purchase sources was undertaken. This was done to address the difficulties associated with clearly identifying tax evasion from tax avoidance in self-report surveys. Such difficulties include socially desirable responding, the prevalence of “under-the-counter” sales from legitimate sources, and counterfeit tobacco that smokers may be unaware of purchasing.^34,38–40^ Sources that are easily accessible to the majority of UK smokers were contrasted with sources where arguably an effort was made to obtain cheap tobacco. The former was considered to be “UK store-based” sources (e.g., supermarkets, convenience stores, and tobacconists) and the latter to be “non-UK/nonstore” sources (e.g., duty-free, overseas, Internet, and informal sellers). An “other” category captured the remaining sources, which represented less than 0.52% of all responses (see Figure 1). Keeping these uncertainties in mind, it was nevertheless expected that within the non-UK/nonstore category, “outside the UK” and “duty-free” were likely to be tax avoidance and “informal sellers” and “from friends/relatives” to be tax evasion.

#### Tobacco Price

Price per stick (FM cigarettes: all waves; RYO: available only from Wave 4 in 2005, onwards) was also derived from the last purchase. Based on available data from the most recent six waves (2006–2014), the average grams of tobacco per RYO cigarette for this UK sample was calculated to lie between 0.45 and 0.55 g (data not shown), which is consistent with other research.^41–43^ The mid-point of 0.50 g was chosen as the amount of tobacco per cigarette for calculating price per stick for RYO smokers. Depending on whether the last purchase was FM-P, FM-C, or RYO (see Figure 1), a further series of questions determined how many cartons/packs were purchased, how many packs per carton, how many cigarettes per pack, or the number and weight of RYO pouches purchased. Participants then had the option to report the price of a single unit (one carton, pack, or pouch) or the total paid if they had purchased multiple units. This was then divided by the number of cigarettes or 0.50 g of tobacco purchased. Since price calculations relied on these multiple responses, the occurrence of inconsistencies and missing data was increased. The following improbable responses (determined a priori by consensus among the authors) were therefore excluded from price analyses: (a) all prices per FM stick or 1.0 g of RYO tobacco over £0.50 (prior to Wave 6) or £0.80 (Wave 6 onward); (b) prices per FM stick or 1.0 g of RYO tobacco from UK store-based sources below £0.07 (prior to Wave 6) or £0.10 (Wave 6 onward); and (c) FM packs from UK store-based sources reported to contain more than 50 cigarettes. For comparability over time, all prices were converted into 2014 values using Consumer Price Index (CPI) data.

The median reported price for each product type last purchased within each source was calculated, and for UK store-based sources only, the “price range” for each product type was also calculated. The price range was truncated to lie between the 2.5th and 97.5th percentiles (capturing 95% of all prices) in an attempt to obtain a more representative value by excluding the most extreme low-frequency cases.

### Analyses

The aim was to examine general population trends in cheap tobacco use, rather than perform predictive modeling. The main results therefore comprise the population-weighted proportion of smokers at each wave using cheap tobacco, and purchasing from various sources, without controlling for any covariates. Multilevel logistic regression analyses using Generalized Estimating Equations (GEE) was also conducted to test for linear trends in proportions over time. GEE estimates population-averaged effects and controls for correlated responses from the same individual over multiple time points. These analyses used a binomial distribution with a logit link function and an unstructured correlation matrix (or exchangeable when the unstructured failed to converge). GEE was also used to test for linear trends in the prices paid for each product type within each purchase source, via multilevel linear regression analyses using a Gaussian distribution with an identity link function. GEE analyses have commonly been used with the ITC data.^14–17,33^

#### Missing Data and Attrition

Missing data for most of the variables used were minimal (see Table 2). However, the price paid for the last purchase could not be calculated for a relatively large proportion of participants due to missing data (2.3–15.2% per wave) or exclusion due to improbable responses (1.0–3.7%). Chi-square analyses were therefore used to compare the group who were excluded from price calculations to the rest of the sample on their responses to the remaining variables. This showed that in a majority of the 10 waves, the price missing group was significantly (*p* < .05): less likely to purchase by the pack rather than the carton or RYO (all waves); less likely to usually smoke exclusively FM cigarettes (9 of 10 waves); less likely to report their purchase source (9 of 10 waves); less likely to report purchasing from UK store-based sources (6 of 10 waves); and less likely to disclose their income (5 of 10 waves). This pattern of responses suggests that the price missing group was more likely to be using cheap tobacco. Estimates of tobacco price, particularly from non-UK/nonstore sources, are therefore likely to be slightly overestimated, and this should be taken into consideration when interpreting results on price.

Participants who had been included in any one survey year were excluded from the analyses at subsequent years if they were lost to follow-up (25.5% of all valid participants), had quit smoking (8.0%), or had missing data (0.6%).

## Results

The prevalence of RYO use increased significantly, and the majority of purchases were from UK store-based sources. There was little change observed in the real prices of cigarettes over the survey period from 2002 to 2014.

### Type of Product (Usual Tobacco Product and Product Last Purchased)

Usual tobacco product type showed a significant linear increase in exclusive RYO use over time (Table 3), with the main increase occurring between 2002 and 2010 and then plateauing. Simultaneously, exclusive FM use significantly declined such that by 2014, only 55% were smoking exclusively FM, 30% RYO, and 15% smoking a mix. The linear trend for mixed FM and RYO use was not statistically significant, although the increase from 10.2% in 2010 (*±*95% confidence interval [CI] = 7.2% to 13.1%) to 18.2% in 2013 (*±*95% CI = 15.3% to 21.2%) coincided with the plateauing of exclusive RYO use. Consistent with this, based on the last purchase data, purchases of FM cigarettes (by the pack and carton) declined significantly as purchases of RYO increased over the survey period.

**Table 3.** Usual tobacco product, product last purchased, and source of last purchase (weighted data), with tests for linear trends over time.

	w1 2002	w2 2003	w3 2004	w4 2005	w5 2006	w6 2007	w7 2008	w8 2010	w9 2013	w10 2014	Β (95% CI) for time trends^a^
Usual tobacco product											
**Factory made only %**	**69.6**	**68.0**	**68.8**	**67.2**	**62.6**	**62.2**	**61.5**	**57.7**	**53.5**	**55.0**	−0.044*** (−0.054 to −0.034)
Last purchase = usual %	82.6	94.1	91.4	95.2	89.6	89.4	92.9	93.9	94.3	93.2	0083*** (0.055 to 0.111)
**Roll-your-own only %**	**17.5**	**19.3**	**20.3**	**22.5**	**25.0**	**26.5**	**24.7**	**30.0**	**28.3**	**30.3**	0.057*** (0.042 to 0.072)
Last purchase = usual %	78.9	90.2	86.7	98.3	90.1	95.0	94.2	95.5	87.1	95.9	0.104*** (0.060 to 0.149)
**Mixed %**	**12.9**	**12.5**	**10.9**	**10.2**	**12.4**	**11.2**	**13.7**	**10.2**	**18.2**	**14.5**	**0.014 (−0.002 to 0.030**)
Last purchase = usual %	69.1	66.0	75.4	81.7	73.2	78.3	73.8	83.8	77.1	83.0	0.048** (0.016 to 0.080)
Product last purchased											
**Factory-made Pack %**	**59.4**	**58.0**	**58.3**	**57.1**	**52.4**	**47.6**	**51.6**	**45.2**	**49.0**	**45.9**	−0.034*** (−0.044 to −0.023)
UK, store-based	97.0	97.2	97.5	97.3	99.2	98.9	98.9	98.9	96.8	98.2	N/A
Non-UK/nonstore	2.6	2.7	2.5	2.6	0.8	0.8	1.1	1.1	2.7	1.1	N/A
**Factory-made Carton %**	**21.4**	**20.5**	**20.2**	**19.6**	**17.8**	**18.4**	**17.1**	**16.7**	**15.2**	**16.9**	−0.039*** (−0.04 to −0.025)
UK, store-based	55.8	45.1	49.8	54.5	54.3	50.2	57.9	61.5	70.7	55.0	0.025* (0.003 to 0.048)
Non-UK/nonstore	44.1	54.9	50.2	45.5	45.7	49.8	42.1	37.3	27.4	44.3	−0.032** (−0.054 to −0.009)
**Roll-your-own %**	**17.3**	**19.0**	**20.7**	**22.5**	**29.5**	**30.0**	**30.5**	**32.2**	**33.2**	**35.0**	0.066*** (0.053 to 0.080)
UK, store-based	71.7	64.9	68.1	65.6	69.4	78.1	66.7	74.2	82.9	80.0	0.045** (0.016 to 0.074)
Non-UK/nonstore	27.9	34.5	31.3	34.0	30.0	21.7	33.3	25.8	16.8	17.6	−0.048** (−0.06 to −0.019)
Source of last purchase											
**UK, store-based %**	**83.7**	**79.9**	**81.6**	**81.8**	**82.4**	**83.3**	**81.5**	**83.2**	**87.1**	**83.7**	**0.019 (−0.002 to −0.041**)
Convenience store	51.2	49.7	50.5	46.4	45.0	50.1	48.0	44.2	40.0	41.0	−0.033*** (−0.044 to −0.022)
Supermarket	41.4	42.4	44.5	49.8	50.6	47.4	47.9	52.4	54.1	54.2	0.040*** (0.028 to 0.051)
UK, store-based other^b^	7.4	7.9	5.0	3.9	4.4	2.6	4.0	3.4	5.9	4.8	−0.038** (−0.066 to −0.010)
**Non-UK/nonstore %**	**15.9**	**19.6**	**18.3**	**18.1**	**17.4**	**16.1**	**17.9**	**16.5**	**11.5**	**14.3**	−0.025** (−0.041 to −0.009)
Outside the UK	55.6	52.9	53.2	43.5	41.1	57.6	28.8	33.6	37.7	39.5	−0.077*** (−0.11 to −0.048)
Duty-free	25.8	27.6	27.8	34.7	34.1	21.8	36.8	35.1	32.4	36.3	0.044** (0.015 to 0.072)
Informal sellers	16.1	16.4	7.2	6.6	5.9	5.8	13.9	7.8	11.5	6.4	−0.081** (−0.14 to −0.027)
Friend/relative	2.2	0.4	9.8	13.9	15.2	14.2	19.3	23.6	11.5	13.4	0.12*** (0.086 to 0.14)
Non-UK/nonstore other^b^	0.2	2.7	2.0	1.3	3.7	0.6	1.2	0.0	6.9	4.4	N/A
**Other, %**	**0.4**	**0.5**	**0.1**	**0.2**	**0.2**	**0.6**	**0.6**	**0.3**	**1.4**	**2.0**	**N/A**

^a^Tests for trend were not conducted (N/A) when floor or ceiling effects were apparent.

^b^“UK store-based other” category includes discount stores, tobacconists, bars/entertainment venues, off-licenses, vending machines, and unclassified UK store-based responses; “non-UK/nonstore other” category includes military commissaries, toll-free numbers, internet purchases, refusals to answer and unclassified non-UK/nonstore responses.

^*^ *p* < .05. ^**^p < .01. ^***^p < .001.

For all smokers (FM, RYO, and mixed), there was a significant linear increase over time in the proportion who reported that their last purchase was their usual brand (see Table 3). Among exclusive FM or RYO smokers, the proportion last purchasing their usual brand was generally high (above 90% in most waves), whereas for mixed users it was somewhat lower, ranging between 66.0% (*±*95% CI = 58.0% to 74.1%) in 2003 to 83.8% (*±*95% C.I. = 74.9% to 92.8%) in 2013.

### Source of Last Tobacco Purchase

UK store-based sources accounted for 80% or more of all purchases (Table 3), and this proportion did not vary significantly over time. The majority of UK store-based purchases were from convenience stores and supermarkets. Within this group, however, there was a significant decrease in purchases from convenience stores and a corresponding increase in supermarket purchases over time such that by 2014, significantly more purchases were from supermarkets (54.1%: *±* 95% CI = 50.0% to 58.1%) than convenience stores (40.0%: *±*95% CI = 36.0% to 44.0%).

Purchases from non-UK/nonstore sources showed a significant downward linear trend over time. It is therefore unexpected that purchases from UK store-based sources did not show a statistically significant linear increase but remained relatively stable over time. This is attributable to the proportion with missing data on source (see Table 2). These participants were arguably more likely to have purchased from non-UK/nonstore sources, because smokers with missing data on price had a pattern of missing data consistent with using cheap tobacco (including missing data on source). When all missing data were assumed to be non-UK/nonstore purchases, the linear trends were no longer statistically significant. Whichever way the missing cases are categorized, there was no indication that non-UK/nonstore purchases were increasing overall.

Within the group purchasing from non-UK/nonstore sources, purchases from outside the United Kingdom were most common (40% or more in most waves) but declined significantly over time, with the largest drop occurring between 2007 and 2008. Duty-free purchases showed a significant linear increase with time, reaching 36% in 2014. Purchasing from informal sellers was below 17% in all waves and significantly decreased over time. Purchasing from friends or relatives was initially very low but increased significantly over time, reaching a peak in 2010 at 23.6%. Online and phone purchases combined accounted for less than 7% of all non-UK/nonstore purchases over the survey period.

At least 97% of FM-P purchases were from UK store-based sources (Table 3). These consistently high figures constituted ceiling effects and precluded statistical tests for linear trend. Relatively fewer FM-C purchases were from UK store-based sources (between 45.1% and 70.7% over the survey period). However, these increased significantly over time, whereas FM-C purchases from non-UK/nonstore sources significantly declined. This suggests that the overall decline in FM-C purchasing noted earlier was largely due to a decline in non-UK/nonstore sources. Finally, the proportion of RYO purchases made from UK store-based sources increased significantly over time, whereas those from non-UK/nonstore sources declined.

### Tobacco Price

#### UK Store-Based Sources

**Figure 2.**
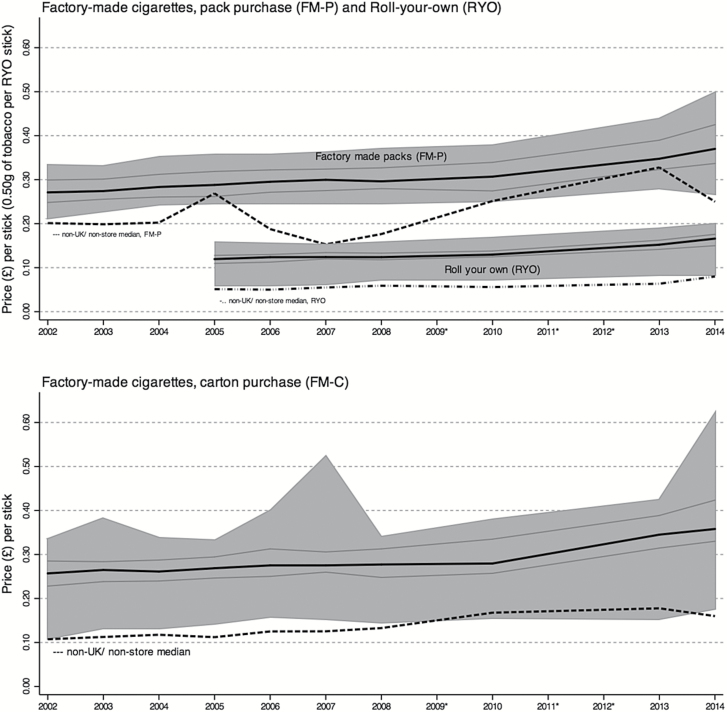
Weighted median price over time of tobacco for factory-made packs, factory-made cartons, and roll-your-own tobacco purchased from UK store-based sources (bold lines) and non-UK/nonstore sources (dotted lines). Shaded area represents 95% of all prices (excluding minimum and maximum 2.5%) from UK store-based sources and is indicative of the price range. Light lines represent the 25th and 75th percentiles. All prices are adjusted to 2014 values and based on the most recent purchase. *Indicates no data collected for these years.

Significant linear increases over time were observed in the real prices of all tobacco product types from UK store-based sources. Prices remained essentially unchanged up to 2010, however, then increased slightly thereafter (Figure 2). In real terms, the median price per stick for FM cigarettes (both pack and carton purchases) rose by only 10 pence over the entire 12 years of the study. Median prices per stick for FM-P rose from £0.27 in 2002 to £0.37 in 2014, with FM-C typically one or two pence cheaper per stick. For RYO, the real median price per stick (0.50 g) increased by only 5 pence over the 9-year period for which data were available, from £0.12 in 2005 to £0.17 in 2014.

The price *range* for tobacco products purchased from UK store-based sources varied by product type (Figure 2), but across all three it was possible in 2014 to buy the same type of product at real prices similar to 2002. The range for FM-P was relatively narrow and changed little between 2002 (£0.12 per stick) and 2010 (£0.13), followed by an increase to £0.16 in 2013, and then a more marked widening to £0.23 in 2014, where both an increase in the highest price and a decrease in the lowest price was evident. For FM-C, although median prices were similar to FM-P, the range was wider and more variable, ranging between £0.19 and £0.27 in most years, with spikes of £0.37 in 2007 and £0.45 in 2014. The price range for RYO tobacco changed little over the period measured, from £0.10 in 2005 to £0.12 per 0.50 g stick in 2014. It may be seen in Figure 2, however, that for FM-P, the prices were evenly distributed over the range, whereas for RYO they were negatively skewed such that there was greater variation at the cheaper (below median) end of the price range.

#### Non-UK/Nonstore Sources

The FM-P from non-UK/nonstore sources accounted for a very small portion of tobacco purchases (see Table 3), so there were limited data available to calculate precise estimates of median prices per stick, and trend analyses were not conducted. As may be seen in Figure 2, however, median prices were consistently at least £0.02 below that of FM-P from UK store-based sources, although the fluctuations were large. More consistent price estimates were obtained for FM-C, which were often less than half the price of UK store-based sources, and changed little (£0.11 per stick in 2002 and £0.16 in 2014), although this increase was statistically significant. Similarly, median prices for non-UK/nonstore RYO tobacco were considerably cheaper than UK store-based sources, ranging from £0.05 per 0.50 g stick in 2005 to £0.08 in 2016, again a significant linear increase. Median prices from non-UK/nonstore sources were often cheaper than the cheapest products available from UK store-based sources for all tobacco product types (see Figure 2).

## Discussion

This article shows there are numerous options for UK smokers to minimize their tobacco expenditure, thus mitigating the public health impact of tobacco tax/price increases. They do so largely by purchasing cheap products from UK stores (84% purchased from cheap sources in 2014). Significant increases in exclusive RYO use and declines in exclusive FM cigarette use were observed. A considerable proportion of smokers (15% in 2014) were identified who usually smoked both RYO and FM products, and this group appeared to be the least brand loyal. Purchasing FM by the carton was not uncommon, with around one in six smokers choosing to buy FM cigarettes in bulk this way. We found no evidence to support industry arguments that smokers are increasingly engaging in tax avoidance or evasion, insofar as this is captured in this study by non-UK/nonstore sources. The analysis of real prices supports these findings. There were clear price incentives to “down-trade” both between and within products purchased from the legal market (UK store-based sources). Hence although for all three product types (FM-P, FM-C, and RYO) the median price from non-UK/nonstore sources was typically lower than the cheapest products from UK store-based sources, it was possible to purchase all three types, legally, at prices below their 2002 medians.

Although the trading of illicit tobacco products from UK store-based sources cannot entirely be ruled out, the present findings suggest tax avoidance and evasion are not the predominant source of cheap tobacco. A change in the most common *source* of tobacco purchases in the United Kingdom occurred during the study, from convenience stores to supermarkets, which would have conferred price savings. Changing product *type* also enabled considerable savings. For example, FM-C purchases typically conferred a saving of £0.01 to £0.03 per stick on the median price, compared to FM-P. A larger saving could be made by switching to RYO, a 0.50-g stick being typically around £0.18 cheaper than one FM-P cigarette: less than half the price. For the average UK smoker smoking around 11.4 cigarettes per day,^44^ this is a saving of about £750 per year compared to smoking FM-P. Changing *within* product types also led to savings. The price range between the cheapest and the most expensive FM-C products was consistently wide, and from around 2010 onward, the price range of FM-P products also widened markedly, providing more opportunities to switch to cheaper brands. The tobacco industry pricing strategy of overshifting tax increases on premium FM brands to maximize profits while undershifting to maintain lower prices for discount FM brands has been observed worldwide.^10,15,19,45,46^ The present findings indicate that this strategy is becoming more aggressive in the United Kingdom. Unlike for FM-P, the observed price range for RYO was disproportionately due to more variation in the cheap (below median) prices. This suggests that industry undershifting may be particularly relevant within the RYO market, a finding not previously observed.

Purchasing from non-UK/nonstore sources did not increase overall (even when we assumed all missing data on purchase source to be non-UK/nonstore) and the nonsignificant trends were for a decrease. However, some more specific trends are worth highlighting. The majority of non-UK/nonstore purchases were duty-free or from outside the United Kingdom, with purchasing from duty-free sources increasing significantly over time and non-UK purchases declining. There were few reports (typically under 25% of non-UK/nonstore purchases and under 5% of all purchases) of sources most likely to be tax evasion, such as informal sellers or from friends or relatives. Purchasing from informal sellers significantly decreased over the survey period, whereas purchasing from friends or relatives increased, reaching a peak in 2010 at just under a quarter of all non-UK/nonstore purchases. Online and phone purchases combined accounted for less than 7% of all non-UK/nonstore purchases over the survey period. The FM-C was most commonly purchased from non-UK/nonstore sources, followed by RYO.

The economic recession that occurred in the United Kingdom in the last quarter of 2008 appeared to influence tobacco purchasing patterns. Around this time, purchases from supermarkets overtook convenience stores as the most popular purchase source, and there was also a drop in purchases made outside the United Kingdom and a spike from informal sellers and friends or relatives. Government policies have also played a role. For example, the period from 2011 onward where tobacco duty was higher than in previous years (at 2–5% above inflation) coincides with the more accelerated increase in tobacco prices from UK store-based sources observed in the present study. Even if this relationship was causal, however, the tax increases did not have a substantial impact on prices in real terms, had no apparent effect on the widening gap between the cheapest and most expensive FM-P products, and little impact on the lowest price paid for RYO. Overall, when inflation is taken into consideration, although statistically significant, the increase in the median price paid for tobacco between 2002 and 2014 was not substantial. On the other hand, the UK strategies to reduce illicit tobacco supply and use appears to have been successful.^30,47^ Taken together, purchases from sources that would most likely represent tax evasion and avoidance did not increase.

### Policy Implications

While efforts have been made in the United Kingdom in recent years for higher tax increases on RYO than those of FM,^24^ considerable price differentials remain. We echo the call from previous UK researchers^18^ for larger relative tax increases for RYO to reduce the price differentials, a move that is likely to result in a reduction in RYO consumption.^16^ In order to further address the tobacco industry practice of undershifting tobacco prices on FM cigarettes, the UK government has committed to introducing a Minimum Excise Tax (MET) in 2017, which will help to raise the price of the cheapest FM tobacco brands.^48^ The exact value of the MET is not yet known, and careful observation will be required to determine if it is sufficient. For maximal impact, the MET should be at least equivalent to the amount of tax currently due based on the weighted average price of tobacco.^49^ Alternative measures such as price-cap regulations^50^ and moving toward a fully specific tax structure^13,15^ may better address this problem. The sale of FM cigarettes in cartons could also be banned, the limits on duty-free purchases could be further reduced or removed altogether, and cross-border purchases could be limited, given the close proximity of the United Kingdom to countries with cheaper tobacco. The current rate of increase of 2% above inflation for UK tobacco excise taxes could also be raised to strengthen impact. Combining this with the other measures outlined could help to increase price while reducing price differentials and the availability of cheaper tobacco.

### Further Research

Purchases from friends or relatives have remained at rates significantly higher than what was observed at the start of the study period. More research is needed to determine whether this constitutes tax avoidance and/or tax evasion or an effort to pool resources in order to buy in bulk. Subpopulations of particular interest are mixed smokers of both RYO and FM cigarettes and also those who do not consider themselves to be brand loyal. These groups may be particularly susceptible to tobacco price changes and warrant further exploration. Our research team is currently exploring the socioeconomic and addiction-related factors associated with cheap tobacco use to assist in the development of more targeted price-based smoking intervention strategies.

### Limitations

The survey included a considerable period (2011 and 2012) where no data were collected, precluding the observation of any fluctuations specific to this period, and perhaps overestimating the linear nature of the trends over time. The large, longitudinal sample of smokers does, however, allow for the observation of overall patterns with some confidence. It is not possible to definitively pinpoint instances of tax evasion in self-reports, as in this study and described in detail elsewhere,^16^ and this may explain discrepancies with UK government estimates.^51^ In particular, “under-the-counter” purchases from legitimate sources cannot be ruled out. This is unlikely, however, as reports of very low prices paid from UK store-based sources were excluded in an effort to overcome this issue. The continuing availability of very low-priced tobacco products from UK store-based sources may be due to a deepening of discounting, increased tax-evasion, or both. Framing the problem in terms of UK store-based versus non-UK/nonstore sources, however, has the advantage of contrasting ease of access for the majority of UK smokers with directed efforts to buy cheaper. If cheap tobacco is increasingly available from the most accessible sources, then this is something that requires careful monitoring, whether or not it is due to tax evasion.

To be included in the study, participants had to be current smokers, so our procedure meant that quitters were progressively excluded from the analysis. However, the missing data analysis also suggested that users of cheap tobacco (who are less likely to quit^11–13,31^) were more likely to be excluded. Thus, these two effects balance each other to some extent. The ITC survey is replenished at each wave with a representative sample of current smokers, tominimize attrition effects. We therefore think it is unlikely that the observed trends are due to attrition.

## Conclusion

UK smokers have many options to reduce their tobacco expenditure and largely do so by purchasing from UK stores. Wide price ranges for each product type (FM-P, FM-C, RYO) from UK store-based sources in 2014 meant that smokers could buy the same products legally at prices comparable to 2002, 12 years prior. Price differences between product types also drove switching from FM to RYO or from FM-P to FM-C. Several policies that could mitigate these trends have been highlighted.

## Funding

This project was funded by the National Institute for Health Research Public Health Research (project number 13/43/58). The views and opinions expressed therein are those of the authors and do not necessarily reflect those of the Public Health Research programme, NIHR, NHS or the Department of Health. The ITC project is funded by the following grants: Canadian Institutes of Health Research (57897, 79551, 115016), Robert Wood Johnson Foundation (045734), Cancer Research U.K. (C312/A326, C312/A6465, C312/A11039, C312/A11943), Commonwealth Department of Health and Aging, Canadian Tobacco Control Research Initiative (014578), National Health and Medical Research Council of Australia (265903, 450110, APP1005922), U.S. National Cancer Institute (P50 CA111236, R01 CA100362), Ontario Institute for Cancer Research (Senior Investigator Award).

## Declaration of Interests

*AG, RH, SH, AM, and TP are members of the UK Centre for Tobacco & Alcohol Studies, a UK Clinical Research Collaboration Public Health Research: Centre of Excellence whose work is supported by funding from the Medical Research Council, British Heart Foundation, Cancer Research UK, Economic and Social Research Council, and the National Institute for Health Research under the auspices of the UK Clinical Research Collaboration (MR/K023195/1).*
